# Treatment accuracy of standard linear accelerator-based prostate SBRT: the delivered dose assessment of patients treated within two major clinical trials using an in-house position monitoring system

**DOI:** 10.3389/fonc.2024.1372968

**Published:** 2024-08-09

**Authors:** Sankar Arumugam, Tony Young, Catherine Jones, David Pryor, Mark Sidhom

**Affiliations:** ^1^ Department of Medical Physics, Liverpool and Macarthur Cancer Therapy Centres and Ingham Institute, Sydney, NSW, Australia; ^2^ South Western Clinical School, University of New South Wales, Sydney, NSW, Australia; ^3^ Department of Radiation Oncology, Princess Alexandra Hospital, Brisbane, QLD, Australia; ^4^ Department of Radiation Oncology, Liverpool and Macarthur Cancer Therapy Centres, Sydney, NSW, Australia

**Keywords:** prostate SBRT, intrafraction motion, online monitoring, delivered dose assessment, protocol compliance

## Abstract

**Background and purpose:**

The purpose of this study was to assess the dosimetric improvements achieved in prostate stereotactic body radiotherapy (SBRT) treatment within the PROMETHEUS and NINJA trials using an in-house real-time position monitoring system, SeedTracker.

**Methods and materials:**

This study considered a total of 127 prostate SBRT patients treated in the PROMETHEUS (ACTRN12615000223538) and NINJA (ACTRN12618001806257) clinical trials. The SeedTracker position monitoring system was utilized for real-time position monitoring with a 3-mm position tolerance. The doses delivered to the clinical target volume (CTV), rectum, and bladder were assessed by incorporating the actual target position during treatment. The dose that would have been delivered without monitoring was also assessed by incorporating the observed position deviations.

**Results:**

Treatment with position corrections resulted in a mean (range) CTV D99 difference of −0.3 (−1.0 to 0.0) Gy between the planned and delivered dose. Without corrections, this difference would have been −0.6 (−3.7 to 0.0) Gy. Not correcting for position deviations resulted in a statistically significant difference between the planned and delivered CTV D99 (*p* < 0.05). The mean (range) dose difference between the planned and delivered D2cc of the rectum and bladder for treatment with position corrections was −0.1 (−3.7 to 4.7) Gy and −0.1 (−1.7 to 0.5) Gy, respectively. Without corrections, these differences would have been −0.6 (−6.1 to 4.7) Gy and −0.2 (−2.5 to 0.9) Gy.

**Conclusions:**

SeedTracker improved clinical dose volume compliance in prostate SBRT. Without monitoring and corrections, delivered dose would significantly differ from the planned dose.

## Introduction

Prostate cancer is the second most common cancer among men, according to GLOBOCAN 2020 (1). More than 60% of prostate cancer patients undergo radiotherapy as part of their treatment (2). Dose escalation has been shown to improve local control and reduce biochemical failures in the treatment of localized prostate cancer (3). Brachytherapy, whether as a boost or monotherapy, has proven to be an effective approach for treating localized prostate cancer by delivering an escalated dose to the prostate compared to conventional external beam radiotherapy (4, 5). However, the resources and specialized skillset required limit the availability of this technique to a few specialized centers.

Stereotactic body radiotherapy (SBRT) has emerged as a viable alternative to brachytherapy for localized prostate cancer, demonstrating equivalent treatment outcomes in both boost and monotherapy regimens. Initially, most of the clinical evidence for prostate SBRT was established using dedicated CyberKnife treatment platforms (6–9). Advancements in radiotherapy delivery techniques, such as volumetric modulated arc therapy (VMAT), flattening filter-free delivery, and in-room imaging capabilities, have enabled the widespread adoption of prostate SBRT on general purpose gantry-based linear accelerators. In SBRT, tight margins between the clinical target volume (CTV) and planning target volume (PTV) are used to limit high-dose delivery to the rectum and bladder. This necessitates monitoring and correcting prostate position deviations during treatment delivery due to intrafraction motion. However, due to the lack of intrafraction position monitoring capabilities in gantry-based linacs, the earlier prostate SBRT implementations were limited to the availability of additional position monitoring systems/approaches such as implanted radiofrequency transponders-based tracking or implanted fiducial tracking using in-room stereoscopic imaging systems (10, 11).

Many research groups have investigated the feasibility of using kV x-ray imaging systems, available on gantry-based linacs for pretreatment cone-beam computed tomography (CBCT) image-based position verification, for real-time position monitoring (12–15). Our group has developed one such approach and a software system called SeedTracker, which utilizes x-ray images acquired from the XVI imaging system on Elekta linear accelerators to monitor and correct prostate position deviations using implanted intraprostatic gold fiducial markers (15–17). This system has been in clinical use since 2015 in two Australian radiotherapy centers for prostate SBRT patients participating in the PROMETHEUS (PROstate Multicenter External beam radiotherapy Using Stereotactic boost) and NINJA (Novel Integration of New prostate radiation schedules with adjuvant Androgen deprivation) clinical trials. PROMETHEUS is a Phase 2 multicenter trial evaluating a high-dose SBRT boost to the prostate in combination with fractionated external beam radiotherapy (18), while NINJA is a Phase 3 randomized clinical trial comparing the prostate SBRT boost regimen with SBRT monotherapy (19).

In this study, we aim to assess the improvement in treatment delivery accuracy in prostate SBRT utilizing SeedTracker for patients treated within the PROMETHEUS and NINJA trials. The study will consider the impact of SeedTracker on treatment, as well as the positional and dosimetric variations.

## Methods

### Patient data

This study included a total of 127 prostate patients who received SBRT and had gold fiducial markers implanted. These patients were part of two separate clinical trials that utilized SeedTracker during treatment. Specifically, 54 prostate patients were involved in the PROMETHEUS trial (ACTRN12615000223538), and 73 prostate patients were included in the NINJA trial (ACTRN12618001806257). The CTV included the prostate and proximal 10 mm of seminal vesicles. Patients with cancer invasion in seminal vesicles were excluded from both trials. All patients had VMAT treatment plans created using dual full arcs with a gantry spacing of 4°. The pre-treatment patient position was verified using CBCT by aligning the implanted gold fiducial markers in the reference planning and verification CBCT image sets. **Table 1** presents patient characteristics and dose prescriptions, while **Supplementary Table S1** shows key treatment planning compliance parameters for both clinical trials.

**Table 1:** The key characteristics of patient and plans considered in the study.

Key characteristics		Clinical trial		
		PROMETHEUS	NINJA	
			Arm 1	Arm 2
Number of patients (*n*)		54	36	37
Age (years)	Median	67	67	
	Range	53–80	51–78	
CTV (cc)	Median	48.9	40.7	
	Range	19.9–100.2	16.9–75.6	
Prostate–rectum separation aid		Rectafix (*n* = 10) SpaceOAR (*n* = 43) None (*n* = 1)	SpaceOAR	
CTV to PTV margin (mm)		3 mm posterior, 5 mm in all other directions	Uniform 3 mm	
Dose prescription		20 Gy in 2#	40 Gy in 5#	20 Gy in 2#

### Online monitoring

Prostate online position monitoring was conducted using SeedTracker, a position monitoring software developed in-house. This software utilized planar x-ray images acquired during treatment to verify the position of the implanted fiducial markers in the prostate. The centroids of the fiducial markers and isocenter co-ordinates from the DICOM RT plan and Structure files were used as the reference data set for the SeedTracker system. During real-time monitoring, the SeedTracker reads the x-ray images acquired during treatment and auto-segments the fiducial markers using a Marker Enhancement Filter (15). The centroids of the auto-segmented markers, corrected for gantry angle-specific imager arm flex, are compared against the planned position to determine position deviations, comparing them to the planned positions. A tolerance of 3 mm was applied to the fiducial marker positions, and the system alerted treatment staff if the markers moved beyond this tolerance. More detailed technical information about SeedTracker can be found elsewhere (15–17). In the event of position deviation, the treatment was interrupted by the user manually. Position corrections were then performed by determining 3D position offsets using a stereo imaging method and treatment was resumed following application of position corrections to the treatment couch [16]. The number of treatment interruptions, when the prostate fiducial makers moved outside the 3-mm tolerance, was recorded for each fraction and assessed. Additionally, the magnitude and direction of deviations was assessed.

### Treatment time

The treatment time data for individual fractions of the studied patients were obtained from the Mosaiq Record and Verify (R&V) system. The difference in treatment time between uninterrupted and interrupted treatment fractions was then analyzed.

### Delivered dose assessment

To assess the dose delivered to target volumes and organs at risk (OARs), the study incorporated the target positions determined by the SeedTracker system using the voxel-shift method (20–22). The dose delivered with the applied position corrections, which took into account the position deviations below the action threshold, was evaluated (referred to as “corrected”). This evaluation involved integrating these deviations into the 3D dose distribution of the VMAT arc for each treatment fraction.

On the other hand, the dose that would have been delivered without monitoring (referred as “not corrected”) was assessed through the following steps:

- In treatment fractions where position deviations did not occur, the residual position errors were incorporated into the VMAT arcs similar to the corrected scenario.
- In cases where position deviations occurred after the CBCT-based patient positioning, but prior to the start of the treatment, the observed position deviation was incorporated into the entire treatment fraction.
- In instances where position deviations occurred during the delivery of the treatment, the residual error calculated up to the fraction of treatment delivery was incorporated into the 3D dose distribution of the control points (CPs) of the VMAT arc up to the gantry angle of the position deviation event. For the remaining dose of the treatment fraction, the magnitude of the position deviation that triggered the event was incorporated into the remaining CPs dose of the VMAT arc.

The dose from individual fractions was summed together rigidly using the DICOM frame of reference of the reference planning dose. To evaluate the accuracy of the planned dose delivery to the target volumes, the CTV D99 (dose delivered to 99% of CTV) and PTV D95 (dose delivered to 95% of PTV) were considered. For the OARs, bladder, rectum, urethra, and nerve bundles, the D2cc, V16 (volume receiving 16 Gy), and V32 (volume receiving 32 Gy) were compared, depending on the clinical trial and fractionation. The CTV D99 was used to assess the variation in minimum dose to the target whilst for the OARs the D2cc was used to study the high dose variation to the OARs. The assessment of target and OARs dose accumulation in this study assumes a rigid relationship of shape and position of the assessed structures with respect to the fiducial markers as in the planning image data set.

### Statistical analysis

The statistical significance of the differences in target volumes and OARs dose between the corrected and not corrected treatment scenarios, compared to the planned dose, was assessed using analysis of variance (ANOVA) and Tukey’s honestly significant difference (HSD) test. A statistical significance level of *p* < 0.05 was used.

## Results

### Treatment interruption and position correction events

**Figure 1:**
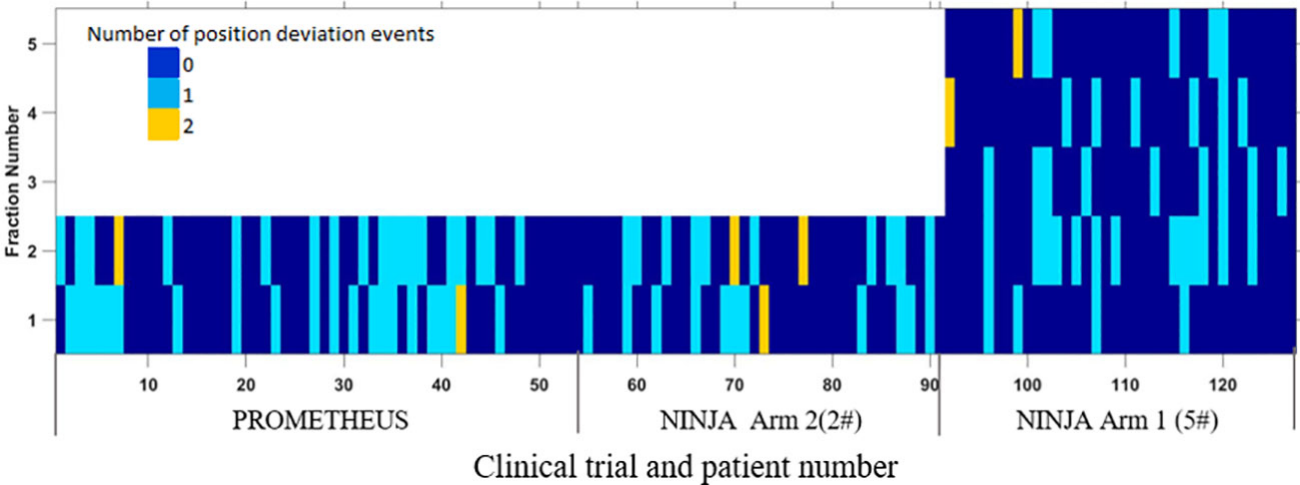
The position deviation in each of the treatment fractions of patients treated in PROMETHEUS and NINJA trials.

**Figure 1** shows the position deviations observed in each of the treatment fractions of patients treated within the PROMETHEUS and NINJA trials. The summary of characteristics and statistics of the position deviation events observed during the treatment delivery is shown in **Supplementary Table S2**.

### Position deviations

**Figure 2:**
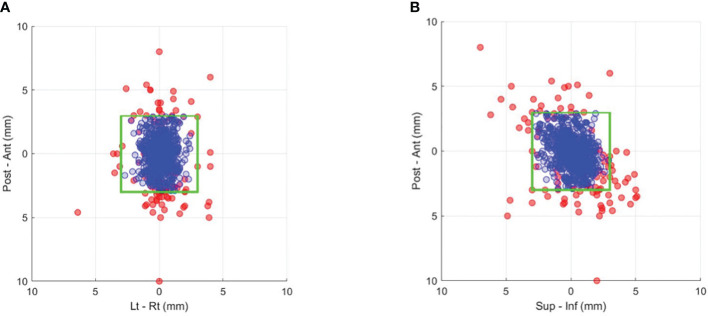
Prostate position that resulted in gating events of position deviations exceeding a 3-mm tolerance (red dots) and the actual corrected position during treatment after applying couch corrections (blue dots) in the **(A)** anterior–posterior and left–right and **(B)** anterior–posterior and superior–inferior directions. The green box indicates the boundary of the 3-mm position tolerance in each direction.

**Figures 2A** and **B** show the prostate position in the AP-LR and AP-SI orientation during treatment (blue circles). The comparison of SeedTracker real-time position data and Mosaiq R&V treatment field record showed that all events during treatment where the target exceeded the real-time position tolerance were interrupted and corrected for. The magnitude of position deviations that resulted in the treatment interruption and position corrections are also shown in the same figures (red circles). With a 3mm position tolerance, an average of 0.3 position deviation events per fraction was observed (**Supplementary Table S2**). Reducing the tolerance to 2 mm would result in 0.57 position deviation events per fraction. Among the detected position deviation events, 36.3%, 35.4%, and 28.3% occurred at the start, during, and before the start of the second treatment arc, respectively (**Supplementary Table S2**). The percentage of position deviations in each orientation, and the maximum magnitude of position deviation is shown in **Supplementary Table S3A**. Also, the direction of position deviation in each orientation is shown in **Supplementary Table S3B**.

### Treatment time

**Figure 3:**
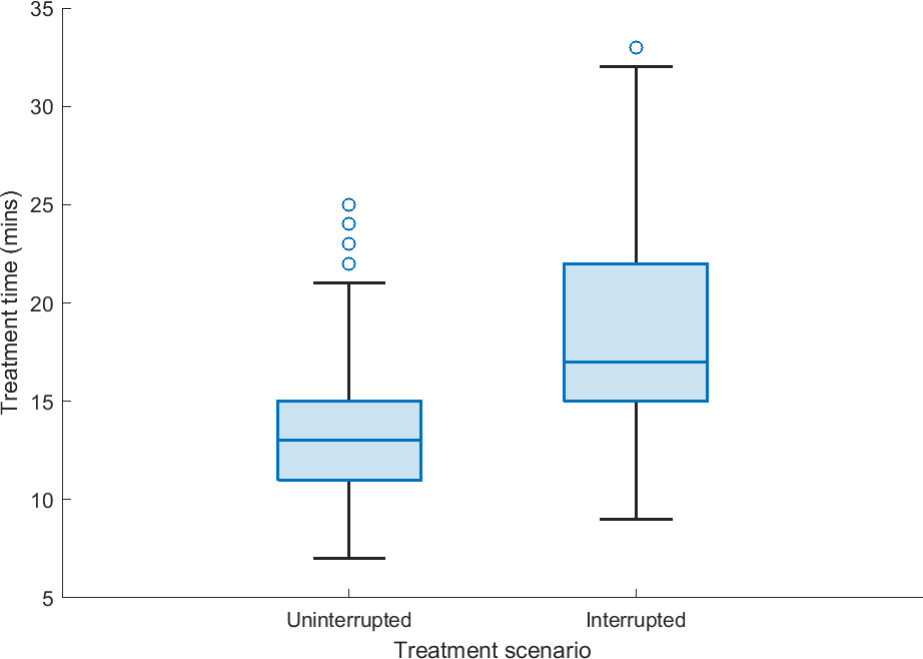
The treatment delivery time of uninterrupted fractions (prostate position within 3-mm position tolerance) and interrupted fractions (prostate position exceeding 3-mm position tolerance, the treatment beam is manually interrupted, position correction was undertaken, and treatment resumed) based on treatment data derived from the Mosaiq record and verify system.

**Figure 3** displays the treatment time for uninterrupted and interrupted treatment fractions in the studied patient cohort. There was a mean increase of 5.5 min for treatment fractions with interruptions compared to uninterrupted treatment fractions where position deviations did not occur.

### Dose assessment

The percentage of plans complying with the clinical goals stipulated by the trial protocol as planned, and treatment with and without position correction scenarios are shown in **Table 2**. The treatment with SeedTracker based real-time monitoring and position corrections resulted in improvement in the compliance of target and OAR dose in both the PROMETEHUS and NINJA plans. Treatment without monitoring and position corrections would have resulted in a high percentage of patients treated with major violations in both coverage to the target and increased dose to the bladder and rectum.

**Table 2:** The key clinical goals compliance of plans and treatment delivery with and without position corrections.

Trial and structures	Metric	% Plans comply with the protocol								
		Planned			Delivery with corrections			Delivery without corrections		
		PP	MiV	MaV	PP	MiV	MaV	PP	MiV	MaV
PROMETHEUS										
CTV	D98	96	4	0	91	9	0	76	17	4
PTV	D95	100	0	0	96	4	0	81	17	2
	D99	100	0	0	100	0	0	87	7	6
Rectal Wall	V16	89	11	0	91	7	2	87	6	7
Bladder	V19	50	22	28	52	24	24	50	22	28
NINJA Arm 1										
CTV	D95	89	11	0	83	17	0	56	44	0
PTV	D95	94	6	0	89	11	0	53	47	0
	D98	97	3	0	86	14	0	89	36	3
Rectum	V40	100	0	0	100	0	0	100	0	0
	V32	100	0	0	100	0	0	100	0	0
Bladder	V40	89	6	6	92	6	3	92	8	0
	V32	100	0	0	100	0	0	97	3	0
NINJA Arm 2										
CTV	D95	97	3	0	86	14	0	68	38	3
PTV	D95	97	3	0	86	14	0	57	27	16
	D98	100	0	0	95	5	0	100	0	0
Rectum	V20	100	0	0	100	0	0	97	0	3
	V16	81	19	0	81	14	3	92	5	3
Bladder	V20	100	0	0	97	3	0	86	8	5
	V16	100	0	0	100	0	0	100	0	0

The clinical goal and thresholds for minor and major violations are presented in **Supplementary Table S1**.

PP, per protocol; MiV, minor violation; MaV, major violation.

#### Target dose

**Figure 4:**
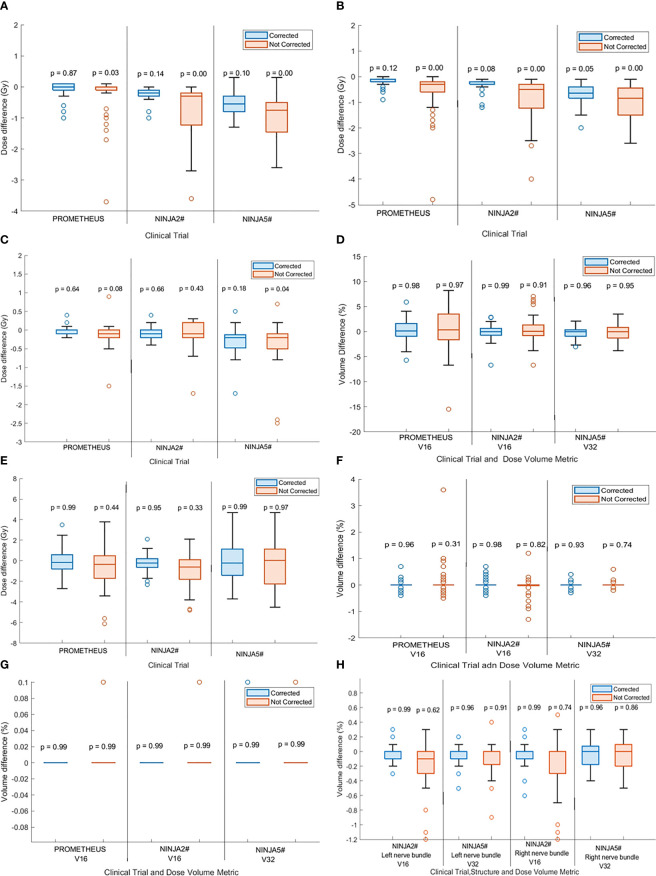
The difference between the planned and delivered **(A)** CTV D99 and **(B)** PTV D95 of treatment with corrected and not corrected scenario along with *p*-values calculated by one-way analysis of variance (ANOVA) and Tukey’s honestly significant difference (HSD) test. The *p* < 0.05 indicates statistically significant difference between planned and delivered dose. The difference between the planned and delivered bladder **(C)** D2cc and **(D)** V16 [for two fractions stereotactic body radiotherapy (SBRT)] and V32 (for five fractions SBRT) of treatment with corrected and not corrected scenario and with *p*-values calculated by one-way ANOVA and Tukey’s HSD test. The difference between the planned and delivered rectum **(E)** D2cc and, **(F)** V16 (for two fractions SBRT) and V32 (for five fractions SBRT) of treatment with corrected and not corrected scenario one-way ANOVA and Tukey’s HSD test. **(G)** The difference between the planned and delivered urethra V16 (for two fractions SBRT) and V32 (for five fractions SBRT) of treatment with corrected and not corrected scenario one-way ANOVA and Tukey’s HSD test. **(H)** The difference between the planned and delivered nerve bundles V16 (for two fractions SBRT) and V32 (for five fractions SBRT) of treatment with corrected and not corrected scenario one-way ANOVA and Tukey’s HSD test.

**Figures 4A** and **B** show the difference between the planned and delivered (corrected) CTV D99 and PTV D95 of patients treated within the PROMETHEUS and NINJA trials. The difference in the target volume dose metrics that would have resulted due to the observed position deviation (not corrected) is shown in the same figure. Additionally, the *p*-value resulting from the one-way ANOVA analysis is presented in the same figures. The application of position correction resulted in consistently reduced differences between the planned and delivered dose in all studied patients. Not correcting for position deviations resulted in statistically significant differences (*p* < 0.05) between the planned and delivered CTV D99 and PTV D95 for patients treated with two fractions; however, with position corrections, there was no statistical difference between the planned and delivered dose (*p* > 0.05). In treatment with five fractions, there was statically significant differences between planned and delivered PTV D95 dose metrics for both corrected and uncorrected scenarios; however, the treatment with position corrections resulted in lower dose differences between the planned and delivered dose (**Figure 4A**).

#### OARs dose

Bladder and rectum:

**Figures 4C** and **D** show the difference in bladder D2cc and V16 (for two fraction treatments) and V32 (for five fraction treatments) between planned and delivered dose with and without position corrections along with the ANOVA statistics for the studied patient cohort treated in both trials. Similar data for the rectum are shown in **Figures 4E** and **F**. There was no statistically significant difference between the planned and delivered bladder and rectum DVH metrics between the corrected and uncorrected treatments; however, the treatment with corrections resulted in consistently smaller differences between the planned and delivered treatments.

Urethra and nerve bundles:

There were no statistically significant differences between planned and delivered V16 (for two fraction treatments) and V32 (for five fraction treatments) for the urethra and nerve bundles for both treatment with and without position corrections (**Figures 4G, H**). Treatment with position corrections resulted in relatively less differences between the planned and delivered V16 and V32 to both right and left nerve bundles in comparison to the treatment without position corrections.

## Discussion

The adoption of prostate SBRT using general purpose linacs is steadily growing. Studies have shown favorable results for prostate SBRT using general purpose linacs when compared to HDR brachytherapy and SBRT using dedicated SBRT machines. While additional real-time position monitoring systems are used with general purpose linacs to achieve the real-time position monitoring, we successfully demonstrated the feasibility of using the XVI imaging system in the Elekta linac in conjunction with an in-house developed software system to perform real-time monitoring for prostate SBRT patients treated within two clinical trials with implanted gold markers.

The continuous monitoring of prostate position and intervention in radiotherapy has been achieved using implanted radiofrequency (RF) transponders previously. In room mounted or integrated stereoscopic x-ray systems are also able to be used to monitor the prostate position within a set time interval. Lovelock et al. reported the utilization of RF transponders for continuous monitoring with a 2-mm position tolerance for linac based prostate SBRT in 89 patients, reporting an average of 1.74 interruptions per treatment fraction (23). In our study, we have reported 0.3 interruptions per treatment fraction, which is much less when compared to the interruptions reported by Lovelock et al. This may be due to Lovelock et al. using a tolerance limit of 2 mm, whereas we have used 3-mm tolerance for the treatment. Further analysis of SeedTracker measured real-time position data with a 2-mm position tolerance resulted in 0.57 interruptions per fraction which is still considerably less than the interruptions reported by Lovelock et al. Additional contributing factors to this difference in treatment interruption frequency may be due to the implementation of a strict bowel preparation protocol and reduced treatment delivery time due to VMAT treatment delivery within our study, compared to the IMRT technique utilized by Lovelock et al. (23).

The direction of prostate motion that triggered the treatment interruption reported in our study qualitatively agrees with other studies that used different position monitoring systems. Shimizu et al. used a RTRT system for intrafraction prostate position monitoring and reported in a cohort of 20 prostate cancer patients treated with a conventional dose fractionation regimen, in 14.2%, 12.3%, and 5.0% of treatment fractions table corrections were required in AP, SI, and LR directions (24). In our study, most position deviation events are triggered by motion in the AP (44%) direction followed by SI (21%) direction (**Supplementary Table S3A**). Based on RF transponder tracking data, Langen et al. reported that the prostate is twice more likely to move inferiorly than superiorly and posteriorly more so than anteriorly. Our data agree with the observations by Langen et al., as the majority of deviations occurred in the posterior (32%) and inferior (20%) directions rather than anterior (20%) and superior (12%) directions (**Supplementary Table S3B**). Bladder filling and bowel movements are common causes of prostate motion in the SI and AP directions. In the majority of our patients a continuous gradual drift of prostate in the inferior direction due to bladder filling was seen, resulting in a position deviation event before the start of second treatment arc and contributed to 28.3% of all interruptions observed.

The dose volume goals to target volumes and OARs specified by the trial protocols were better achieved in treatment delivery with monitoring and correction in both trials. Treatment without monitoring and position corrections would have increased the percentage of minor violations to the CTV D95 to a maximum of 44% of patients treated in NINJA Arm 1, with occurrences of major violations to CTV dose in both PROMETHEUS and NINJA treatments (**Table 2**). With SeedTracker based real-time monitoring and position corrections, the minor violations were reduced to 17% with no occurrences of major violation in Ninja Arm 1 (**Table 2**). While non-compliance in PTV DVH metrics in the delivered dose assessment is expected, a relatively high percentage of major violations were noticed in the NINJA Arm 2 cohort (**Table 2**). A relatively smaller PTV margin (3-mm isotropic) used in this NINJA patient cohort could be the main contributing factor for this. Reducing the position tolerance for real-time monitoring from 3 mm to 2 mm may reduce the PTV D95 noncompliance and potentially reduce the range of differences observed between planned and delivered CTV D99 (**Figure 4A**). PROMETHEUS plans showed a high number of minor (22%) and major (28%) violations of bladder V16. In the PROMETHEUS trial relatively larger PTV margins (5-mm uniform except 3-mm posterior margins) were used for treatment planning and clinicians accepted high V16 to achieve PTV dose constraints. Treatment without position corrections did not increase the percentage of major violations in this studied cohort of patients. For patients treated in NINJA Arm 2, treatment without position correction would have resulted in major violations in 5% of patients. Unlike the CTV and PTV doses, the rectum dose metrics were not affected in the uncorrected treatment scenario. The use of rectal separation aids in both trials likely accounts for this, as the high-dose gradient region falls within the geometric separation between the prostate and rectum. Future studies will investigate the influence of mean prostate-rectal spacing on rectal dose in uncorrected treatment scenarios.

Faccenda et al. studied the dosimetric impact of inter- and intra-fraction position changes of the prostate in 13 prostate SBRT patients using daily CBCT images and real-time prostate position data measured using the RayPilot electromagnetic transponder system (25). They reported statistically significant differences between the planned and delivered CTV D99 and PTV D95 with mean (range) percentage difference of −1.3 (−8.3 to 0.2)% and −1.0 (−5.6 to 0.6)%, respectively. Our study agrees with the results of Faccenda et al., with the delivered dose assessment showing that the D99 to CTV consistently improved with position corrections applied in both two fraction and five fraction SBRT (**Figure 4A**). Not applying position corrections would have resulted in statistically significant differences (*p* < 0.05) between the planned and delivered dose in both the PROMETHEUS and NINJA patient cohorts (18, 19). The CTV D99, which represents the minimum dose received by the CTV, was reduced by a maximum of 3.7 Gy from the planned dose if the position corrections were not performed. The difference between the planned and delivered DVH metrics of OARs was consistently smaller for treatments with position correction in comparison to the uncorrected treatment scenario. A statistically significant difference between the planned and delivered D2cc to bladder was observed if the position corrections were not performed (**Figure 4C**).

The protocol violations and delivered dose discrepancies to both target and OAR volumes without monitoring and position corrections presented in this study emphasize the requirement for continuous monitoring and correction of target position deviations in prostate SBRT. In this study, we have successfully demonstrated the efficacy of SeedTracker, in conjunction with the XVI imaging system on the Elekta linac, in improving prostate SBRT treatments for patients treated within two different clinical trials. To our knowledge, this is the first study that presents the feasibility of utilizing a pre-existing x-ray imaging system for real-time monitoring in multiple clinical trials and on large number of patients (127 patients). The successful demonstration of use of a pre-existing imaging system enables the possibility of widespread adaption of high-precision treatments such as prostate SBRT without the need for additional resources and enables the adoption of high-precision treatment in developing countries where resources may be a challenge.

The dosimetric effect of residual rotational error in the prostate and deformation of target and OAR volumes were not assessed in this study. Wolf et al. studied the dosimetric effect of rotational errors observed in prostate SBRT and found that the CTV dose was not compromised even at a 3-mm CTV-PTV margin due to the sphericity of the prostate volume (26). Maund et al. (27) and Faccenda et al. (25) studied the dosimetric impact of deformations in prostate and OAR volumes using CBCT images for both conventional and stereotactic prostate radiotherapy. Both studies found that the target and OAR volumes dose difference between the original plan and the dose accumulated plan, which accounted for structure deformation, was statistically not significant. While these variations may be considered and accounted for in a daily adaptive planning procedure, it may have less impact on the delivered dose assessment performed in the present study. Ma et al. assessed the dosimetric impact of proximal seminal vesicle shape and position variation in prostate SBRT patients treated on an MRI linac with a 2-mm PTV margin (28). They found that due to significant rotation and volume changes, the volume receiving 95% of the prescription dose (V95) ≥95% was achieved in only 59% of treatment fractions. The patients in our study have relatively larger margins (PROMETHEUS: 3 mm posterior and 5 mm in all other directions, NINJA: 3 mm uniform), and we expect to see relatively improved dose coverage to the seminal vesicles, which are treated adjuvantly in both trials. Future studies will assess the delivered dose to the proximal seminal vesicles of patients treated within the NINJA trial.

## Conclusion

The dosimetric impact and clinical dose volume goals compliance in prostate SBRT patients treated within two clinical trials were studied by incorporating the prostate position determined using the SeedTracker real-time position monitoring system. The delivered dose to the CTV would have been significantly different than the planned dose if monitoring and position corrections were not performed.

## Data Availability

The original contributions presented in the study are included in the article/**Supplementary Material**. Further inquiries can be directed to the corresponding author.
