# Supplementary material for: Treatment accuracy of standard linear accelerator-based prostate SBRT: the delivered dose assessment of patients treated within two major clinical trials using an in-house position monitoring system
Source: Front Oncol. 2024 Aug 9;14:1372968. doi: 10.3389/fonc.2024.1372968 (PMC11341385; doi:10.3389/fonc.2024.1372968)
Supplement: Supplementary file 1 [file Table_1.docx]

Table S1: Summary of key protocol compliance parameters specified in PROMETHEUS and NINJA trial protocols

| Trial | Structures | DVH metric | Compliance | | |
| --- | --- | --- | --- | --- | --- |
|  |  |  | Per Protocol | Minor Violation | Major Violation |
| PROMETHEUS | CTV | D98 | ≥20Gy | 19-20Gy | <19Gy |
|  | PTV | D95 | ≥19Gy | 18-19Gy | <18Gy |
|  |  | D99 | >16Gy | 15-16Gy | <15Gy |
|  | Rectal Wall | V16 | <0.5cc | 0.5-1cc | >1cc |
|  | Bladder | V19 | <10cc | 10-15cc | >15cc |
| NINJA Arm 1 | CTV | D95 | ≥40Gy | 38-40Gy | <38Gy |
|  | PTV | D95 | ≥36Gy | 34.44-36Gy | <34.44Gy |
|  |  | D98 | ≥34.44Gy | 32.72-34.44Gy | <32.72Gy |
|  | Rectum | V40 | ≤0.1cc | NA | >0.1cc |
|  |  | V32 | ≤10% | >10%-20% | >20% |
|  | Bladder | V40 | ≤2cc | 2-3cc | >3cc |
|  |  | V32 | ≤10% | >10%-15% | >15% |
| NINJA Arm 2 | CTV | D95 | ≥20Gy | 18-20Gy | <18Gy |
|  | PTV | D95 | ≥18Gy | 17-18Gy | <17Gy |
|  |  | D98 | ≥17Gy | 16-17Gy | <16Gy |
|  | Rectum | V20 | ≤0.1cc | NA | >0.1cc |
|  |  | V16 | ≤1cc | 1-2cc | >2cc |
|  | Bladder | V20 | ≤2cc | 2-3cc | >3cc |
|  |  | V16 | ≤10% | >10%-15% | >15% |

NA-Not applicable

Table S2: The characteristics of the position deviation events observed during the treatment delivery.

| Parameter | Value |
| --- | --- |
| Total number of treatment fractions monitored | 362 |
| The number of position deviation events per patient | 0.56 |
| The number of position deviation events per fraction | 0.3 |
| Maximum number of gating events observed in the same treatment fractions | 2 |
| Position deviation occurrence timeline during treatment: |  |
| At the start of treatment | 36.3% |
| During the delivery of treatment beam | 35.4% |
| Before the start of second treatment arc | 28.3% |

Table S3a: The percentage of occurrences of prostate position deviations exceeding the position tolerance of 3mm in each orientation.

| Orientation | Percentage of occurrences |  |
| --- | --- | --- |
| Left-Right | 5% | |
| Anterior-Posterior | 44% | |
| Superior-Inferior | 21% | |
| Anterior-Posterior and Superior- Inferior | 18% | |
| Left-Right, Anterior-Posterior and Superior-Inferior | 4% | |
| Left-Right and Anterior-Posterior | 1% | |

Table S3b: The percentage of occurrences of prostate position deviations exceeding the position tolerance of 3mm in each direction.

| Percentage of occurrences of position deviation in each direction | | | | | |
| --- | --- | --- | --- | --- | --- |
| Left | Right | Anterior | Posterior | Superior | Inferior |
| 5% | 8% | 20% | 32% | 12% | 20% |
